# ADAR Family Proteins: A Structural Review

**DOI:** 10.3390/cimb46050243

**Published:** 2024-04-26

**Authors:** Carolyn N. Ashley, Emmanuel Broni, Whelton A. Miller

**Affiliations:** 1 Department of Medicine, Loyola University Medical Center, Loyola University Chicago, Maywood, IL 60153, USA; cashley1@luc.edu (C.N.A.); ebroni@luc.edu (E.B.); 2 Department of Molecular Pharmacology & Neuroscience, Loyola University Medical Center, Loyola University Chicago, Maywood, IL 60153, USA

**Keywords:** ADAR, protein structure, RNA editing, structure-based drug design, deamination

## Abstract

This review aims to highlight the structures of ADAR proteins that have been crucial in the discernment of their functions and are relevant to future therapeutic development. ADAR proteins can correct or diversify genetic information, underscoring their pivotal contribution to protein diversity and the sophistication of neuronal networks. ADAR proteins have numerous functions in RNA editing independent roles and through the mechanisms of A-I RNA editing that continue to be revealed. Provided is a detailed examination of the ADAR family members—ADAR1, ADAR2, and ADAR3—each characterized by distinct isoforms that offer both structural diversity and functional variability, significantly affecting RNA editing mechanisms and exhibiting tissue-specific regulatory patterns, highlighting their shared features, such as double-stranded RNA binding domains (dsRBD) and a catalytic deaminase domain (CDD). Moreover, it explores ADARs’ extensive roles in immunity, RNA interference, and disease modulation, demonstrating their ambivalent nature in both the advancement and inhibition of diseases. Through this comprehensive analysis, the review seeks to underline the potential of targeting ADAR proteins in therapeutic strategies, urging continued investigation into their biological mechanisms and health implications.

## 1. Background

RNA editing was first established in 1986 and is a valuable method for increasing the complexity of proteins by way of insertions, deletions, or base conversions that occur post-transcriptionally [1,2,3]⁠. RNA editing occurs throughout the body but has crucial implications in the central nervous system (CNS) where RNA editing is most prevalent [4]⁠. Deamination events are key forms of RNA editing, including cytodine to uridine (C-U) editing carried out by cytidine deaminases like APOBEC-1 and adenosine to inosine (A-I) editing carried out by adenosine deaminases like a family of proteins called ADARs for adenosine deaminases acting on RNA [2,5,6]⁠. This paper will focus on ADARs as enzymes critical for A-I editing, the most common form of RNA editing that occurs in vertebrates [7].

The ADAR family is made up of three members, ADAR1, ADAR2, and ADAR3. ADAR1 was the first ADAR enzyme discovered in *Xenopus laevis* shown to display deamination capabilities for A-I editing [8,9,10]⁠. ADAR1 and ADAR2 are capable of catalyzing the hydrolytic deamination reaction that converts adenosine into inosine, but ADAR3 has not displayed this activity and instead seems to affect RNA editing in a regulatory manner [11]⁠. The A-I deamination events carried out by ADAR proteins are pivotal in numerous roles including generating protein diversity, but ADARs also have RNA editing independent roles. For example, ADAR1 can form a complex with Dicer that impacts miRNA processing [12]⁠. RNA editing activity/efficiency modulation or mutations affecting ADAR RNA editing independent roles are commonly associated with disease such that understanding RNA editing regulation and mechanisms is important for many fields of discovery, including oncology, virology, and neurology. The full scope of ADAR functions remains unclear, yet the current knowledge of their mechanisms in the innate immune system, CNS disease pathophysiology, and their possibilities for precision medicine make this protein family captivating drug targets and proteins of interest in the development of future therapeutics.

ADAR mechanisms and regulations are crucial in gaining insights into numerous fields of study, and as potential drug targets, understanding the structures and key residues involved in ADAR RNA editing is imperative. Yet, no full structures of ADARs have been published. Unfortunately, the complexity of the pathways and numerous processes ADARs take part in make therapies targeting ADARs potentially dangerous because there is a risk of targeting one pathway and affecting another process with severe adverse effects. This leads to the reason that in order to develop safe therapies targeting ADARs, there is a requirement for more details on which pathways are involved, what structures are involved, and key mutants with therapeutic potential. There have been prodigious steps in these areas. Already many types of therapies and usages for ADAR proteins are coming to light, and ADAR structures and their key residues and elements will be incredibly valuable for the discovery of future targeted therapies. The intent of this paper is to highlight the crucial discoveries of ADAR structures with the aim of describing known critical residues and potential structural targets of ADARs themselves for future drug design and discovery.

First, this review will briefly describe some of the intricacies of ADAR functions as ADARs can both inhibit and promote different diseases. It will become clear that ADAR RNA editing relies on multifaceted mechanisms for elaborate regulation, and when these mechanisms are disturbed, there are biologically relevant consequences in terms of disease pathophysiology. This paper will summarize key aspects of ADAR evolution focusing on pertinent relatives that have aided in the understanding of ADAR structure and the importance of ADAR in the maturation of neurological pathways. ADAR editing regulation is affected not only by alterations to gene expression but also by ADAR variants between tissue types, changes in subcellular localization, effects on RNA transcript specificity or preference, and protein-protein interactions. ADAR domain structures each have important isolated functions and contribute to synergistic functions relevant to these regulatory mechanisms. This paper will cover an in-depth look at the conserved structures and how those structures may impact ADAR editing and regulation, as well as, the member-specific ADAR domains and how they influence ADAR editing and regulation. Lastly, this paper will briefly conclude by describing some ongoing investigations and future objectives in the ADAR field as they relate to the design of ADAR therapies.

## 2. Overview of the Biological Impacts of ADAR

The functions of ADARs as RNA editors cover a wide range but are generally thought to repair miscoded genomic information or diversify the encoded information for higher-level functions [13]⁠. The complex pathways ADARs play a part in are not fully understood, and their biological roles only seem to grow more vast. It is well known that A-I editing can lead to new protein structures, changes in stability, or variations in translation efficiency or splicing [14]⁠. ADARs have demonstrated roles in the innate immune system, the inhibition of RNA interference pathways, modulating miRNA processing and functionality, and the sequestration of RNAs in the nucleus [14,15,16,17,18]⁠. The effects of A-I editing in coding regions have been well documented in neural pathways and can directly impact the functions of the proteins they edit [14]⁠. A-I recoding in the subunits of AMPA and kainate glutamate receptors control crucial functions of these subunits including calcium permeability and receptor desensitization. For example, decreased ADAR2 editing in the GluA2 subunit of the AMPA receptor results in increased calcium permeability and past a threshold, neurodegeneration associated with sporadic amyotrophic lateral sclerosis (ALS) [19,20]⁠. However, the vast majority of A-I editing in humans occurs in noncoding regions, specifically those with inverted Alu repeats where the significance is not fully elucidated [14,21,22]⁠. One functional role of RNA editing within these noncoding regions is in regulating the nuclear export of mRNAs where ADAR A-I editing correlated to the nuclear retention of RNAs with inverted repeated Alu elements [23]⁠.

ADARs clearly play other valuable roles as well. Phenotypes of ADAR2^−/−^ mice experienced epileptic-like seizures and death in early development [24]⁠. ADAR1^−/−^ mutations were embryonically lethal resultant of stress-induced apoptosis and defective proliferation and differentiation of hematopoietic cells [25]⁠. Mice with a deletion of the third exon of ADAR3 display abnormal behaviors associated with anxiety and result in loss of hippocampus-mediated memory formation [26]⁠. The expression of ADARs in the brain and neurons supports that the gain of ADARs greatly contributed to the complexity of the metazoan neuronal network. ADAR1 and ADAR2 are ubiquitously expressed, though ADAR1p110 has noticeably higher levels of expression [27]⁠. ADAR2’s expression is highest in the brain, bladder, and lungs [27]⁠. ADAR3’s expression remains restricted to certain brain regions largely in the hippocampus, thalamus, amygdala, and olfactory region [26]⁠ Neurotransmission is strictly regulated with a requirement for high levels of protein diversity and highly complex protein interaction networks. ADARs are essential for the proper functionality of an assortment of neuronal-related targets and pathways [28,29,30]. Mutations of ADARs or changes in their expression have been connected to various complications including neurological disorders, metabolic disorders, many types of cancer, and viral infections [31,32,33,34,35]⁠. A few of the best-characterized links to ADAR include autoimmune disorders like Aicardi-Goutières syndrome (AGS) and Bilateral Striatal Dystonia (BSD), neurological disorders like Sporadic ALS and epilepsy, and behavioral and psychiatric disorders like Major Depressive Disorder (MDD) and suicidal schizophrenia [36,37,38,39,40]⁠.

**Figure 1:**
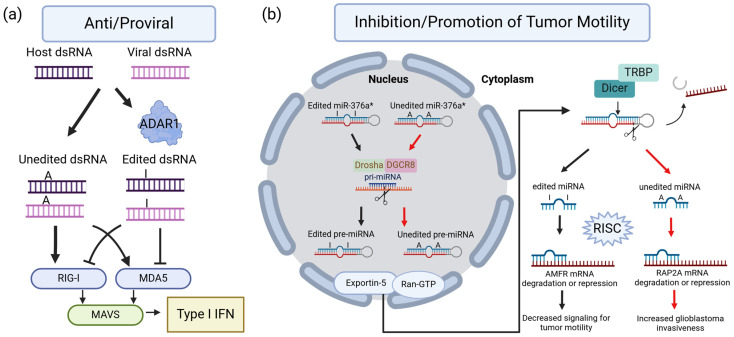
(**a**) Summary cartoon depicting ADAR1’s role in type I interferonopathies. ADAR-edited dsRNA inhibits retinoic acid-inducible gene I (RIG-I) and melanoma differentiation-associated protein 5 (MDA5). Reduction of ADAR editing can result in unedited dsRNA, leading to downstream mitochondrial antiviral signaling proteins (MAVS) complex activation and eventually the expression of type 1 interferon genes [47]⁠. (**b**) Summary cartoon comparing functional outcomes of ADAR-edited miR-376a* and unedited miR-376a*. Notably, ADAR can influence other miRNAs through RNA editing independent roles that impact Drosha or Dicer cleavage, and RNA-induced silencing complexes (RISC) loading [12]⁠. Created with BioRender.com.

ADAR proteins affect different pathways which can allow for both the promotion of disease and protection from disease. This duality of ADARs is most easily explained in cancer and viral infections (Figure 1). ADAR1 is involved in recognizing self versus non-self dsRNA. A-I editing can protect our own dsRNA, and a lack of inosines can be a key identifier of foreign dsRNA. ADAR1-edited dsRNA inhibits MDA5 and Rig1 activation (Figure 1a) [41]⁠. MDA5 and RIG-1 promote MAVS signaling, ultimately leading to the type 1 IFN response (Figure 1a) [41]⁠. However, some viruses like the Hepatitis B virus (HBV) make use of ADAR1 to protect their own dsRNA from immune detection [42]⁠. This pathway can also lead to issues outside of viral infection. ADAR1 editing is associated with AGS, the loss of ADAR1 editing by the decreased expression or less efficient ADAR1 mutants results in an upregulation of type 1 interferon signaling [36]⁠.

ADARs also have a dual role in cancer by being both suppressors and promoters through various pathways. One example of ADAR complexity in cancer is through RNA editing level changes that lead to differential targeting of miRNAs. ADAR editing of pri-miRNAs can lead to changes in mRNA target specificity by editing within the seed sequence, inhibiting Dicer or Drosha-mediated cleavage, and suppressing RISC loading (Figure 1b) [43,44,45]⁠. In glioblastoma cells, the ADAR editing of the miRNA, miR-376a*, influences its targets. Under normal ADAR editing, miR-367a* targets the autocrine motility factor receptor (AMFR), decreasing signals calling for tumor motility (Figure 1b) [46]⁠. However, when miR-367a* remains unedited, it no longer targets the AMFR and instead targets RAP2A mRNA, a suppressor of cell invasiveness in glioblastoma, promoting glioblastoma invasiveness (Figure 1b) [46]⁠. This example aims to highlight how ADAR can alter protein functions influencing disease.

ADAR RNA editing levels are clearly biologically relevant in both normal functions and disease states. The determination of different ADAR domain structures has greatly furthered our understanding of RNA editing mechanisms. Details into the networks controlling ADAR regulation are critical as ADAR RNA editing varies between tissue types, maturation, and throughout disease progression. ADAR gene expression can be altered by multiple processes specific to each ADAR member [48]⁠. However, alterations to gene expression alone do not explain all of the complex variations in RNA editing. For example, a study of psoriasis reported that reduced ADAR2 expression levels correlated with reduced RNA editing. Yet, while ADAR1 RNA editing was also reduced, ADAR1’s expression was upregulated [49]⁠. Mechanisms other than expression level control alone must be involved in RNA editing regulation. This review will cover some of the studies looking at the structures and functions of individual ADAR domains that have identified other mechanisms that affect ADAR regulation including ADAR variants, subcellular localization, RNA transcript specificity or preference, and protein-protein interactions.

## 3. ADAR Evolution

**Figure 2:**
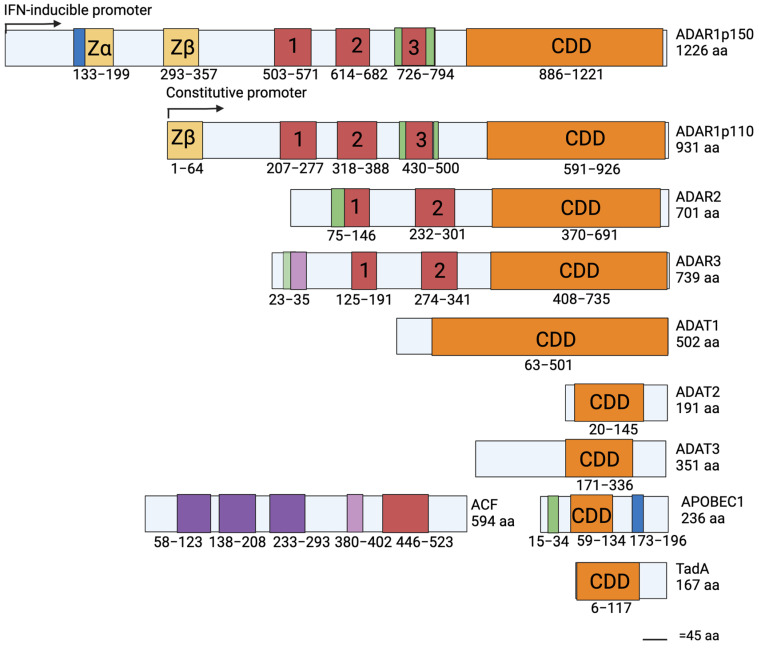
Domain architectures of the main ADAR isoforms, ADAT1, ADAT2, ADAT3, APOBEC1, APOBEC1 Complementation Factor (ACF), and TadA. The color code is as follows: yellow for the Z-DNA-binding domains, blue for NES, green for NLS, red for dsRNA-binding domains, orange for the catalytic domain, pink for the Arginine-rich domain, and purple for RNA recognition motifs. The information for these architectures was found using UniProt accession numbers: P55265 (ADAR1), Q9NS39 (ADAR3), Q9BUB4 (ADAT1), Q7Z6V5 (ADAT2), Q96EY9 (ADAT3), P41238 (APOBEC1), Q9NQ94 (ACF), and P68398 (TadA), NCBI reference sequence NP_001103.1 (ADAR2) and published data from the following sources [50,51,52]⁠. Created with BioRender.com.

**Figure 3:**
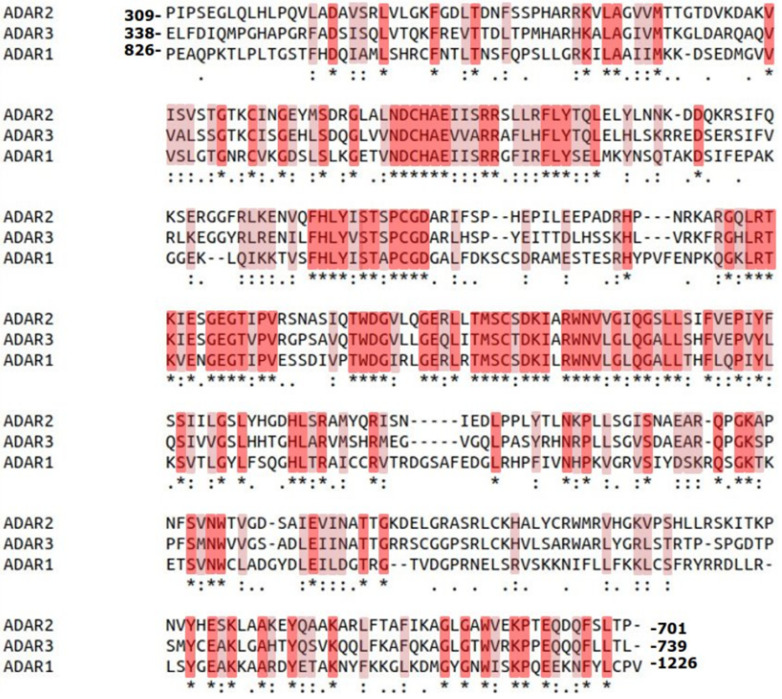
Sequence alignment of the catalytic deaminase domain (CDD) of ADAR2, ADAR3, and ADAR1. Highlighted in red with asterisks are completely conserved residues, highlighted in pink with colons are residues that are conserved between groups of amino acids with strongly similar properties, and white with periods correspond to conservation between groups of amino acids with weakly similar properties. Sequences were obtained from UniProt ADAR1p150 (ID_P55265) and ADAR3 (ID_Q9NS39), and NCBI: ADAR2 (ID_NP_001103.1). Multiple sequence alignment was performed using Clustal W.

The four main isoforms of ADAR include ADAR1p150, ADAR1p110, ADAR2, and ADAR3 (Figure 2). All ADAR members contain two common structures, double-stranded RNA binding domains (dsRBD) and a catalytic deaminase domain (CDD) (Figure 2). ADAR1 contains three dsRBD; ADAR2 and ADAR3 contain two. Structural knowledge has demonstrated how these conserved domains influence ADAR editing and other functions such as through alteration of specificity for transcripts. Additionally, ADARs have some domains that are distinctive of certain members. For example, ADAR3 contains an arginine-rich domain (R domain) that does not appear in the other family members. In looking at sequence alignments of ADAR proteins (Figure 3), they have high sequence identity and similarities. ADAR3 is most similar to ADAR2 at 56% sequence identity and 75% sequence similarity. ADAR1 has 39% identity to both ADAR2 and ADAR3 and has sequence similarities of 55% and 54% to ADAR2 and ADAR3, respectively. As ADAR proteins have very high sequence similarity, knowledge of their conserved structures contributes to overall ADAR knowledge, and identifying these proteins’ unique structures will be insightful in understanding their member-specific functions.

Studies detecting ADAR family members across many species of early sponges and ctenophores indicate that the ADAR proteins in humans are likely similar to the ADARs existing in the last common ancestor of the metazoan lineage [13,53,54]⁠. ADARs have not been detected in *Monosiga brevicollis,* the closest unicellular relative to metazoans, nor in plants, yeast, fungi, or prokaryotic genomes [53,54]⁠. Though ADARs were lacking prior to the crown metazoan, A-I editing itself spans further back in evolutionary history. The A-I mechanism has been observed in prokaryotes through the bacterial tRNA adenosine deaminase (TadA), an ortholog of the adenosine deaminase acting on the tRNA 2 (ADAT) family [55]⁠. ADATs and the conversion of A-I editing in tRNA are found in all eukaryotes. ADAT1 functions similarly to ADARs, making a compelling case for their evolution and they have key residues involved in catalytic activity. ADAT1 deaminates adenosine in the tRNA wobble position to inosine, however, ADAT1 differs structurally as it only contains a CDD [53,56]⁠. In contrast to ADAT1, ADAT2 and ADAT3 form a heterodimer and ADAT3 is considered inactive [57]⁠. Evolutionary relatives thought to prelude ADARs main branch from two protein groups: adenosine deaminases acting on mononucleotides (ADA) and cytidine deaminases acting on mononucleotides (CDA). It is the CDA group that is thought to preclude other RNA editing proteins like APOBEC members, ADATs, and even prokaryotic orthologs like TadA [53,54]⁠.

ADAR has predicted relation to these proteins because of the presence of conserved residues important for their catalytic activity, similar structures, and functional domains. The original crystal structure of the isolated CDD of ADAR2 depicts the catalytic core which can be split into zinc coordinating residues and a buried basic cavity lined with arginine and lysines containing the negatively charged inositol-hexakisphosphate (IP6) [58]⁠. The catalytic center has four major residues: E396 which H-bonds the nucleophilic water molecule which is activated by the zinc ion arranged by residues H394, C451, and C516 (Figure S1) [58]⁠. These four residues are crucial for deamination and also remain conserved in CDA proteins and TadA [59,60]⁠. Additionally, the presence of IP6 in the CDD is essential to ADAR catalytic activity. The necessity of the IP6 binding has been useful for potential ADAR therapies where the IP6 binding cavity has been used as a target site for small molecule inhibitors that can be used to potentially regulate ADAR2 [61]⁠. These coordinating residues for IP6 remain largely conserved between ADAR1, ADAR2, and members of the ADAT1 family. ADAT2 members and TadA have no IP6 cavity and lack the conserved residues, whereas ADAR3 inactivity is not related to IP6 loss as it retains the residues for IP6 coordination (Figure 3) [58]⁠.

The catalytic domains of ADATs, ADARs, and APOBECs have similarities supporting that they likely diverged from the same family. Comparing the deaminase domains of ADARs to CDA or ADA further supports that the CDA family proteins likely prelude ADARs [59,60]⁠. APOBEC family proteins have catalytic domains similar to that of ADAR proteins, and the catalytic activity remains reliant on zinc coordination. The residues for the zinc coordination remain conserved between ADAR and APOBEC1 [60]⁠. While APOBEC1 retains a CDD, it does not have dsRBD. APOBEC1 is different from ADARs because it requires protein cofactors like ACF that contain a similar dsRBD for RNA binding (Figure 2) [62]⁠. Between ADARs and ADATs, ADAT1 is closest to the ADAR family. ADARs potentially originated after an event where ADAT1 gained a dsRBD region. Other ADAR members were then likely generated through the following gene duplications, and the eventual gain and loss of domains resulted in the variations of the ADAR gene present across different metazoan species [53]⁠. Specifically, ADAR1 and ADAR2 likely diverged early from this originating ADAR member. Then, as ADAR3 has an extremely high sequence similarity with ADAR2, ADAR3 likely developed resultant of an ADAR2 duplication [53]⁠.

## 4. The Base Flipping Mechanism of ADARs and the Inactive ADAR3

The CDD of ADAR2 has been well described and provided is a table summarizing the key residues and their functional involvement (Table 1). ADAR3, at first glance, shares high-sequence similarity with both ADAR1 and ADAR2 and appears to have the necessary residues and structures required for deamination, yet ADAR3 has notably not displayed deaminase activity (Figure 3) [63]⁠. ADARs carry out the hydrolytic deamination of the amino group located at the C6 position of adenosine for conversion to inosine [64]⁠. The mechanism of deamination requires that the adenosine to be edited be moved out of the duplex RNA helix as shown in Figure S1. The proposal of a base flipping mechanism has been around for a long time and is not distinctive of ADARs, but structures depicting the CDD of ADAR2 in complex with RNA substrate solidified this method [65,66]⁠. This base flipping mechanism works by a base flipping loop (residues 487–489) approaching from the minor groove where residue E488 surrounded by G487 and G489 are inserted and fills the gap that forms from flipping the to-be-edited adenosine out of the helix and into the active site (Figure S1). As ADAR approaches from the minor groove, residues R510 and S495 are important for stabilizing interactions for the distinct manipulations of the RNA that must occur to warp the major groove. Interestingly, R510 is a key residue for interaction with the orphaned base across from the flipped-out adenosine, this residue corresponds to R1032 in ADAR1, but is instead Q549 in ADAR3. Mutations of R510 to Q or A result in a deamination reduction of around one magnitude [65]⁠. ADAR3 inactivity has been previously suggested to arise from a lack of dimerization but may occur in vivo despite the results of in vitro assays [67]⁠. However, isolated ADAR1 CDD and ADAR2 CDD can carry out editing, so if dimerization is required for ADAR3 editing, this would suggest a unique mechanism. Results from a 2019 study suggest the catalytic domain of ADAR3 cannot carry out the deamination reaction despite the fact that ADAR sequence similarities of the catalytic deaminase domain are highly conserved [68]⁠. This study was looking for potential ADAR3 substrates and generated an active ADAR3 CDD capable of deamination activity through the mutation of only five amino acids: A389V, V485I, E527Q, Q549R, and Q733D [68]⁠. E527Q is a mutation corresponding to E488Q in ADAR2, a mutation known to increase catalytic activity. The other four amino acids, A389, V485, Q549, and Q733, are not conserved between ADAR3 and the other ADAR members and represent mutations that must inhibit ADAR3 deaminase activity as the ADAR3 CDD contains the other key conserved residues known to be involved in deamination. This study was carried out in a human glioblastoma cell line but showed that the wt ADAR3 was capable of regulating transcript levels of DUSP1 and EGR1. Additionally, the inactive CDD of ADAR3 may have importance still as mutations like E527K are frequently reported in the Catalogue of Somatic Mutations in Cancer (COSMIC) database [11,69]⁠. ADAR3 has been reported to have a multitude of somatic mutations (3052) within its coding region compared to only 730 and 1033 in ADAR1 and ADAR2, respectively [11]⁠. In mutation experiments, mutations such as E527K and Q549R led to increased RNA binding ability and when testing the effects of these mutations on MAVS exhibited how ADAR3 binding is involved in regulating the protein expression levels of MAVS in an RNA editing independent fashion [11]⁠. Overall, even without deaminase activity, ADAR3 has its own functionality by regulating its own substrates as well as negatively regulating the editing of other ADAR substrates [26,63]⁠. One example of negative regulation is ADAR3 inhibition of ADAR2 editing in *GRIA2* transcripts. ADAR2 editing of *GRIA2* transcripts is decreased in tumors from human glioblastoma patients [70]⁠. In vivo, ADAR3 was able to inhibit ADAR2 editing of the Q/R site mediated through ADAR3’s dsRBDs [71]⁠. The increase in expression levels of ADAR3 in these patients led to deficient editing of the *GRIA2* transcripts by ADAR2. Regardless of the loss of deamination in ADAR3, the conservation of the A-I mechanism and ADAR enzymes in metazoan lineages indicate their functional significance, likely the gain of ADARs promoted diversity and influenced the regulation of RNA imperative to the development of neuronal complexity in metazoans.

## 5. Conserved Structures and Their Effects on Substrate Specificity

There are two structures that are present in all ADAR family members. The first are dsRBDs. Across species of ADAR variants, there can be various dsRBDs; human ADAR1 has three, and human ADAR2 and ADAR3 each contain two. Each ADAR additionally contains a CDD that contains the enzymatic active site. The involvement of both structures in regulating RNA substrate specificity and preference is quite complex. ADAR editing is certainly affected by the shape and length of the duplex RNA where, dependent on the structure and length of an RNA substrate, the form of ADAR editing can be changed. Duplex RNA that is short or broken into segments by structural features like bulges, mismatches, or loops leads to a targeted and accurate form of ADAR editing [72,73]⁠. This site-specific editing can be highly precise, editing the same adenosine out of a stretch of adenosines each time. Another form of ADAR editing is rapid hypermutation editing. Most duplex RNAs that experience this hypermutation are more than 50 base pairs long and perfectly complimentary [73,74]⁠. The structure and length of RNA substrates do not solve all questions about ADAR editing. For example, in a study using the GluR-B pre-mRNA that includes an important R/G editing site, they showed that ADAR2 could selectively edit this transcript, but ADAR1 edited promiscuously [75]⁠. Differences in editing between ADAR1 and ADAR2 are influenced, at least in part, by nearest neighbor preferences, where ADARs may bind any duplex RNA but the bases to either side of an edited adenosine favor particular bases. The 5′ nearest neighbor preferences of hADAR1 are U=A>C>G and ADAR2 are U≈A>C=G, and the 3′ nearest neighbor preference for hADAR2 is U=G>C=A [73,74,75,76]. ADARs also show a preference for the neighbor of adenosine on the complimentary strand where A-C=A-U>A-A and A-G mismatches [76]⁠. The use of isolated ADAR domain structures determined that the CDD controls the nearest neighbor preferences but was indicative of the 3′ preference of ADAR2 being attributed to the dsRBD [77]⁠. The structure of the ADAR2 CDD with substrate provides evidence that the basis for these 5′ preferences amount to a steric clash between unfavored neighbors with residue G489 [65]⁠. While these clashes are a hindrance and do diminish the editing of adenosines within these unfavorable contexts, they do not stop deamination from occurring entirely, hence, only a preference of ADARs for certain neighbors [65]⁠. Similarly, it can be inferred that adenosines with favorable bases as neighbors are considered in good context and are more likely to be edited, which may contribute to the high selectivity of certain sites. This same study provides evidence that the 3′ preference of ADAR2 may also result from the CDD due to a guanine’s ability to provide an H-bond to the carbonyl oxygen of S486 in the CDD [65]⁠. This contrasts with earlier structures depicting the 3′ preferences as a result of the second dsRBD of ADAR2 [78]⁠. Both structures cannot exist at the time but may be indicative of sequential binding and release of ADAR dsRBDs followed by CDD binding. This idea was previously suggested after finding that internal loops in surrounding RNA structures could affect editing efficiency without impacting RNA binding selectivity, indicating a separation between the binding of ADARs to substrates and the deamination of particular adenosines [75]⁠. Other models for the simultaneous binding of both the CDD and dsRBD have been suggested. These models grew upon work using truncated ADAR2 forms containing only the second dsRBD and the CDD which remained catalytically active at the GluR-B R/G site; note that lack of both dsRBD led to a loss of editing at the site, determining the dsRBD is required for this substrate [79]. The concurrent model suggested involves the CDD binding and widening the major groove leading to the formation of a wider A-type RNA groove. Prior structures have revealed the dsRBD ability to bind the A helix in this same conformation [78,80]⁠. The binding of dsRBD2 can also result in major groove widening, suggesting that either CDD or dsRBD may bind first and help render the duplex for binding of the other domain, though this model requires an additional conformational change of the CDD [81]⁠. Certain substrates like the 5HT_2C_R site would not allow for this concurrent model, but other longer substrates or substrates with additional binding sites for the dsRBD could accommodate simultaneous binding [81]⁠.

Accounting for RNA structures, substrate lengths, and nearest neighbor preferences, to explain the incredible efficiency in with certain transcripts are edited requires even more regulation. In experiments where the CDDs of ADAR1 and ADAR2 were swapped, the substrate specificity was correlated to the CDD, indicating that the CDD does affect the specificity of certain substrates [76]⁠. However, the CDD of ADAR1 and ADAR2 have a high-sequence similarity reported to be 39% identical and 59% similar [82]⁠. So which structures in the CDD give rise to these ADAR-specific editing occurrences? There have been many residues determined to be involved in the functionality of the CDD for RNA editing (Table 1). X-ray crystal structures are indicative of an RNA binding loop that may explain the differences in ADAR1 and ADAR2 substrate selectivity. This binding loop in ADAR2’s CDD covers residues 454–477 and becomes ordered upon contact with the RNA minor groove where it is then inserted into the major groove [65]⁠. Between ADAR1 and ADAR2 there are considerable differences in sequence inside this RNA binding loop, demonstrating its potential to affect ADAR specificity [65]⁠. Other residues entirely conserved among ADAR2 sequences include contact residues G593, K594, and R348 that if mutated, drastically decrease deamination [65]⁠. Other residues may influence ADAR1 editing, as ADAR1 has been suggested to have an additional zinc-binding site within its CDD that is not seen in ADAR2 [83]⁠. Key residues of this second zinc-binding site include H988, C1081, C1082, and H1103 which are highly conserved across ADAR1s, but in ADAR2 correspond instead to Y561, Q562, and K578. Mutations of residues C1081, C1082, and H1103 negatively affect ADAR1 deamination in human cells, indicating their importance to ADAR1 [83]⁠. This interaction may be an alternative way to stabilize the ADAR1 protein fold, as the corresponding residues in ADAR2, Y561, Q562, and K578 are used for ADAR2 folding and stability. Overall, it is evident that the CDD does impact substrate specificity, however, the dsRBD does appear to have some impact, as well as other potential functions. Several studies have shown that the dsRBDs participate in ADAR localization, substrate binding affinity, and substrate specificity [84,85]⁠.

**Figure 4:**
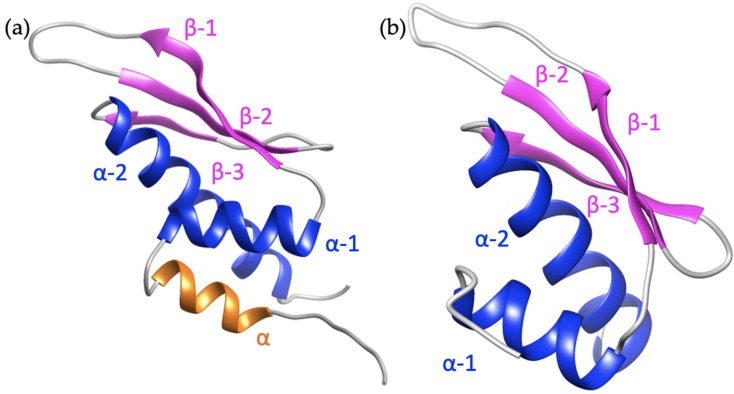
Comparison of dsRBD structures with conserved α helices in blue, β sheets in magenta, and the unique α helix in orange. (**a**) The third dsRBD of ADAR1, PDB: 2MDR, topology ααβββα. (**b**) The first dsRBD of ADAR2, PDB: 2B7T, topology αβββα.

The dsRBDs are not limited to eukaryotes but also present in eubacteria, viruses, and an Archaeon [86]⁠. More evolutionarily advanced organisms acquire multiple copies of dsRBDs, indicating that each may have diverse functions, an idea reinforced by the presence of multiple dsRBDs across many families of proteins with distinct functions [87]⁠. The dsRBD are about ~65 amino acids long and have a conserved topology of αβββα where loops connect each secondary structure (Figure 4). This domain/motif occurs in many RNA binding proteins (RBP) besides ADARs including Staufen [88]⁠, human TAR-RBP [89]⁠, Dicer [90]⁠, and spermatid perinuclear RBP (SPNR) [91]⁠, which are each involved in a variety of roles including host-viral responses, mRNA and RNA regulation and localization, and RNA interference. The major function of dsRBDs is to bind duplex RNA occurring predominantly in a shape-dependent manner. X-ray crystals of the complex between dsRBD and duplex RNA reveal that the method of substrate binding recognition relies on interactions with the dsRBD and the sugar-phosphate backbone, indicating a shape-dependent recognition [80]⁠. In some instances, there are particular contacts in the minor groove that seem to act in a sequence-specific manner [79,81]⁠. As reiteration structures of this readout may not be relevant without the CDD but may suggest a catch and release of the dsRBD before CDD binding. Regardless, canonical binding contacts between dsRBD and RNA substrates include the solvent-faced residues of the first helix, the GPxH motif inside the loop between β1β2, and the KKxAK motif of the second helix [80]⁠. The number of dsRBDs also increases the affinity of ADAR for dsRNA and can increase editing activity [92]⁠. The structures of the first and second dsRBDs of ADAR2 and the structure of the third dsRBD of ADAR1 have been produced (PDBs: 2B7T, 2B7V, and 2MDR, respectively) [79,93]⁠. Based on sequence alignments the first and third dsRBD of ADAR1 fits best with the first and second dsRBDs of ADAR2 (Figure S2). Interestingly, the second dsRBD of ADAR1 is not required for enzymatic activity and may play some other function [94]⁠. The relevance of dsRBDs in RNA-independent functions like protein-protein interactions is steadily growing.

The most coherent function of the dsRBD in protein-protein interactions is their involvement in ADAR localization. Each ADAR is impacted by its subcellular localization. ADAR1p110, ADAR2, and ADAR3 localize to the nucleus, concentrated mainly in the nucleolus, whereas ADAR1p150 is predominantly localized in the cytoplasm [95,96]⁠. ADAR1p150 is the full-length ADAR1 isoform that results from an interferon-inducible promoter, whereas the ADAR1p110 form results from a constitutive promoter. ADAR1p150 contains a CRM1-mediated NES in its Zα domain [95]⁠. The ADAR1p110 isoform is missing the N-terminal NES and Z-DNA binding domain α [10,97]⁠. Present in both ADAR1p150 and ADAR1p110 are flanking portions of an NLS surrounding the third dsRBD [93,98]⁠. This means ADAR1p150 can shuttle to the cytoplasm and back to the nucleus, but ADAR1p110 remains only in the nucleus. The NLS of ADAR1 is coined bimodular in that it consists of two NLS fragments flanking either side of the third dsRBD and the structure of the three dsRBD depicts an extra alpha helix in its N-terminus (Figure 4a) that acts as a scaffold for the two fragments of the NLS to interact with transportin-1 (Trn1) [93]⁠. This interaction of the NLS with Trn1 does not occur while the dsRBD is bound to dsRNA [93]⁠. Instead, nuclear export is dependent on RNA binding of the dsRBD, indicating a regulatory role of the dsRBD in ADAR1 nuclear shuttling. Experimental data confirms that increased RNA binding correspondingly increases the nuclear export [99]⁠. Likely dsRBD3 binds substrates in the nucleus and ADAR is subsequently exported out, and then Trn1 binding in the cytoplasm may increase the dissociation of the dsRNA substrates and lead to the return of ADAR1 to the nucleus [100]⁠. The nuclear export of ADAR1 has potentially multiple methods of occurring. Experimental data reporting the interaction of Exportin-5 (Exp-5) with each of the three dsRBDs of ADAR1p110 occurred reliant on RanGTP or if RNA was bound in a RanGTP-independent manner [99]⁠. An additional NES is reported to exist within the Z dsRBDs [95]⁠. The localization role of dsRBD extends further into chromosomal targeting where experiments show that the three dsRBD alone are capable of chromosomal site recognition in a site-selective manner and even an individual dsRBD can target specific transcriptionally active subsets of chromosomal sites [101]⁠.

Both ADAR2 and ADAR3 contain an NLS in their N-terminus, where the R domain of ADAR3 appears to act as an NLS [102]⁠. ADAR2 movement to the nucleoplasm directly corresponds to increased RNA editing [18]⁠. Mutation experiments within the dsRBD of ADAR2 provide evidence that it is the binding of the dsRBD to substrates (in this case duplexes of rRNA) that control the subcellular localization [18]⁠. These studies also highlight the importance of the conserved lysine residues for RNA binding as it is involved in H-bonding to the phosphodiester backbone and, upon mutation binding, is lost [18]⁠. Completely conserved residues within the ADAR dsRBDs are depicted in Figure S3.

The other incredibly relevant, however, not entirely understood method for dsRBD in protein-protein interactions, involves a major question in ADAR functionality; do ADARs function as dimers? While ADARs are purified as monomers capable of deamination, evidence from both structural and biochemical characterizations postulates that at least ADAR1 and ADAR2 can form dimers. The similarity of the ADAR CDD with that of *Escherichia coli* cytidine deaminase, cytodine deaminase APOBEC1, and ADATs gave rationale early on for dimerization as the cytodine deaminases and members ADAT2 and ADAT3 require dimerization for enzymatic activity [103,104,105]⁠. Other proteins with motifs similar to the ADAR dsRBDs like Staufen, PKR (dsRNA-dependent protein kinase), and RNase III likewise dimerize using their dsRBDs indicative that the dsRBD may play a role in ADAR dimerization [106,107,108]⁠. Many studies have supported ADAR dimerization as either homodimers or heterodimers. In a kinetic study, ADAR2 monomeric cross-linking was seemingly required for productive editing supporting potential ADAR2 homodimerization [109]⁠. Another study reported an RNA-independent homodimerization of both ADAR1 and ADAR2 with coimmunoprecipitation results that argued against the formation of ADAR heterodimers [67]⁠. The general consensus, thus, approves of ADAR dimerization, but questions remain. Is dimerization RNA-(in)dependent? That is can ADARs can form dimers without being bound to a substrate, or must they bind to the substrate first in order to dimerize? Some studies support RNA-dependent dimerization that requires dsRBDs for dimerization [110,111]⁠. Other studies support an RNA-independent method of dimerization [12,112,113]⁠. In support of RNA-dependent dimerization, a study using Drosophila ADAR depicted a model where an ADAR monomer would bind duplex RNA first, and following binding, a second monomer would join to form the dimer capable of deamination, which would follow as previously suggested a separation between ADAR binding and catalysis [111]⁠. In this model, only the N-terminus and the first dsRBD were required to form dimers, but intriguingly, mutants without N-terminus residues 1–46 lost their dimerization ability and showed increased affinity for dsRNA, enough to compete with the wt-ADAR [111]⁠. This argues that binding is not related to dimerization, and since dimerization is correlated to deamination activity, suggests that binding and deamination are indeed separate events. However, RNA-dependent interactions are not the only ones seen, ADAR1 homodimers and protein-protein interactions between ADAR1 and Dicer have been discovered which occur in an RNA-independent manner [12]⁠. Similarly, ADAR mutants with nonfunctional dsRBDs are capable of dimerization as have ADAR fusion proteins given RNase treatments [112,113]⁠. It can, therefore, be postulated that since ADARs can form dimers without substrate, perhaps there is some regulation occurring through the formation of these dimers. Logically, an ADAR dimer formed prior to substrate binding would be immediately prepared for deamination upon binding of its substrate. Whereas if an ADAR monomer binds substrate first it must then wait for another monomer or protein subunit to arrive before deamination may occur, which may lead to the site being skipped if the monomer dissociates before deamination occurs. However, isolated CDDs can edit some sites, although with lower efficiency. Adenosine may still be edited by a monomer and that event would be affected by which ADAR variant is bound, the structure and length of the duplex RNA, and the context of its nearest neighbors, along with potentially unaccounted-for interactions.

One suggested regulation may be the combination of different ADAR variants generating ADAR dimers expressing varying levels of editing efficiency or RNA binding that may contribute to altered editing between different cell types both in vitro and in vivo [111]⁠. ADAR2 has a multitude of variants all arising from variation in usage of alternative splice sites [114]⁠. Of the many ADAR2 variants, few have shown distinction from the functionality of the 701 amino acid ADAR2 form, that is the ADAR2 variants mainly maintain similar editing selectivity and efficiency. Although, two ADAR2 isoforms have demonstrated the ability to decrease ADAR2 editing activity. One forms from the inclusion of exon5a, resulting in an Alu cassette addition in the catalytic domain. The other forms as a result of self-editing in the intron 1 resultant of an additional 47 nucleotides to the 5′ end of exon 2 [115,116]⁠. Another ADAR2 isoform of interest contains a sequence motif similar to the arginine-rich domain of ADAR3, and this ADAR2 isoform is produced by initiation at an alternative promoter, resulting in the extension of 49 nucleotides in the N-terminus [102,117]⁠. Still, more research on ADAR2 variants and ADAR tissue-specific regulation is needed. The many variants of ADAR2 support that the multitude of variants play some role in cell and tissue-specific editing divergences [118,119]⁠.

**Figure 5:**
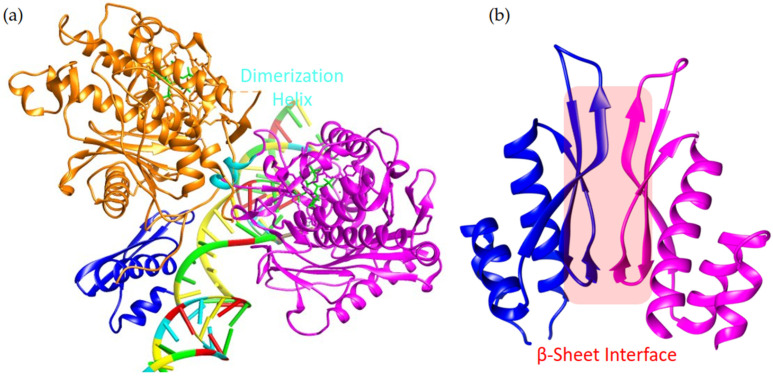
Current dimer models for ADAR2 and ADAR1. (**a**) ADAR2 dimer model, PDB: 6VFF, depicts dimer formation through a dimerization helix (in cyan) between two CDDs of ADAR2 colored orange and magenta, respectively. The magenta CDD binds the RNA substrate (colored with the NDB color scheme), whereas the orange CDD carries out editing. The second dsRBD, colored blue, is connected to the orange CDD and is capable of binding the dsRNA. (**b**) ADAR1 dimer model, PDB: 7ZJ1, depicts ADAR1 dimer formation between monomers of the third dsRBDs of ADAR1 represented in blue and magenta, respectively. The dimer interface occurs between the β-sheets of the dsRBD highlighted in red. Notably, PDB: 7ZLQ not visualized here supports that both dsRBDs in the ADAR1 dimer retain the ability to bind dsRNA.

Dimerization exists as a potential regulation mechanism of ADARs but it remains undetermined where the full dimerization interfaces are located. As mentioned in dADAR, the N-terminus and first dsRBD were required for dimerization. The need for dsRBD is similarly expressed in humans although both the first dsRBD and second dsRBD were involved in dimerization, yet only the second dsRBD was necessary for the editing of the GluR2 Q/R site [110]⁠. Chimeric dimer generation also displayed a necessity for dsRBDs as each ADAR monomer in the chimeras required a functional dsRBD for both binding and editing activity [113]⁠. To date, two different ADAR dimer structures have been assessed (Figure 5). In ADAR2, an X-ray crystal structure has been captured of a partial ADAR2 homodimer (PDB: 6VFF). This structure consists of two monomers each containing the CDD and second dsRBD of ADAR2 bound to RNA substrate GLI1 (Figure 5a) [120]⁠. In ADAR1, a homodimer formation between the 3dsRBD of one monomer to the 3dsRBD of a second monomer, where the dimer interface is located between contacts in the beta sheets (PDBs: 7ZJ1 and 7ZLQ) (Figure 5b) [121]⁠. These two structures each contribute important knowledge about their respective ADAR family member and about dimerization for ADAR editing regulation.

Relevant to the ADAR2 homodimer structure, the combination of the CDD and second dsRBD of ADAR2 can edit substrates with which the isolated CDD is incapable [77]⁠. The first monomer is considered the catalytically active monomer as it has the 8-azanebularine hydrate flipped out into its active site. The second monomer is considered the substrate binding monomer as it does not use its CDD for deamination, but uses its dsRBD to attach 3′ of the editing site likely aiding in RNA binding. This substrate-binding monomer uses segments of its RNA binding surface and catalytic cavity to contact the first monomer via an alpha helix in the first monomer’s CDD spanning residues 501–509 [120]⁠. The dsRBD of the catalytic monomer does appear to interact with RNA, though its electron density was not sufficient to appear in the crystal structure produced. Key residues for the protein-protein interaction between the first and second monomer include conserved residues T501, W502, D503, G504, G508, and L509 which reside in the dimerization helix (Figure 5a) [120]⁠. Mutations of T501, W502, or D503 each lead to varying effects on dimerization, highlighting their influence on the dimerization interaction [120]⁠. Interestingly, ADAR3 also retains the residues involved in the dimerization helix. As ADAR3 has been resolved as a negative regulator of both ADAR1 and ADAR2, it remains feasible that in the brain, ADAR3 may form heterodimers to modulate the editing activities of the other ADARs [63,67]⁠. Other participating residues and their interactions are displayed in Table 2 and for more information on individual contributions, see [120]⁠.

The structure of the ADAR1 symmetric homodimer supports the use of dsRBD3 for ADAR1 homodimer formation. Two parts of ADAR1 are capable of dimer formation, the CDD and the 3dsRBD [12,120,121]⁠. The CDD of ADAR1 can lead to dimer formation, although this interaction is substrate-dependent. In the absence of the CDD, the 3dsRBD of ADAR1 retains the ability to bind dsRNA through its canonical mechanism and dimerization can occur in an RNA-independent manner due to its strong affinity, at least in vitro [121]⁠. The key in this study was the ability to disrupt ADAR1 dimer formation through mutations (V747A, D748Q, W768V, and C773S) [121]⁠. This mutant allowed some insight into the role of dimerization for ADAR1 as disruption of dimerization could reduce ADAR1 editing activity of specific substrates [121]⁠. Also notable was that the ADAR1–3dsRBD dimer did not disrupt the localization of either ADAR1p150 or ADAR1p110 [121]⁠. The key contact residues within the ADAR1 dimer model are provided in Table S1. This ADAR1 symmetric dimer structure in combination with the asymmetric dimer interface of ADAR2 brings up a few more questions about ADARs. In the ADAR2 dimer structure there is an active and inactive monomer, which leads to the question in the ADAR1 structure, would the presence of the CDD together with the 3dsRBD lead to other dimer conformations? In exchange, could other homodimer conformations exist for ADAR2? How do the full-length ADAR proteins impact dimerization/mitigate the effects of dimer disruption? Both ADAR1 and ADAR2 have high sequence similarities, do they form a similar conformation for an ADAR1-ADAR2 heterodimer? Heterodimer formation has been suggested for all three ADAR family members, ADAR1, 2, and 3, and they each have high sequence similarity. So, where do the heterodimer interfaces lie? Could ADAR dimer disruption be a method for ADAR therapeutics? Much is left to be discovered about ADAR dimerization, and since dimerization inhibition can modulate ADAR editing without globally decreasing ADAR editing, there is therapeutic potential here. Overall, full protein structures would greatly expand knowledge for determining relevant dimers with therapeutic potential.

## 6. The Family Member Specific Structures

### 6.1. Z-DNA Binding Domains of ADAR1

Z-DNA was originally discovered in 1979 [122]⁠. Z-DNA differs from the canonical Watson and Crick B-DNA by being a left-handed double helix with an alternating anti-syn base conformation and zig-zagging pattern of the sugar-phosphate backbone [122,123]⁠. ADAR1p150 was the first protein identified to bind Z-DNA and contains two Z-DNA binding domains, Zα and Zβ [124]⁠. The Zα domain is capable of binding both Z-DNA and Z-RNA [125]⁠. Isoform ADAR1p110 contains only the Zβ domain that is incapable of binding Z-DNA or Z-RNA [123]⁠. The Zα and Zβ domains are not entirely required for RNA editing of either RNA or DNA strands as the isolated catalytic domain remains capable of such activities [126]⁠. However, the Zα domain is required for the editing of a particular portion of RNA substrates and is also involved in ADAR1 localization and efficient ADAR1 editing activities [127,128]⁠.

The ability of Zα to bind Z-DNA may be essential for certain substrates because of where Z-DNA forms. Z-DNA is stabilized by negative supercoiling that occurs upstream of active RNA polymerase and is common in sequences with alternating purine pyrimidine sequences [129,130]⁠. Thereby the role of Zα binding to Z-DNA sites on nascent pre-mRNA may explain how ADAR1 is able to localize to actively transcribing sequences and edit mRNAs swiftly before splicing. The Zα domain can also form a stable interaction with ribosomes capable of translation blockage, which may further aid ADAR1 editing of nascent pre-mRNAs [131]⁠. Notably, while ADAR1 has the potential to inhibit translation, in vivo, the binding of ADAR1 to Z-DNA in a promoter region functions to promote transcription [132]⁠.

ADAR1 is involved in distinguishing between self and non-self nucleic acids, regulation of the innate immune system, both proviral and antiviral responses, and both pro and anticancer pathways [16,33,47,133]⁠. One way ADAR1 controls the innate immune response is through suppression of the type I interferon response [134]⁠. Mutations of the Zα domain of ADAR1 result in phenotypes associated with type I interferonopathies suggesting that the disruption of Z-DNA or Z-RNA binding is responsible for disturbed ADAR1 function, resulting in autoimmune disorders like Aicardi-Goutières syndrome (AGS) and Bilateral Striatal Dystonia (BSD) [37,135]⁠. A functional Zα domain is also necessary for the localization of ADAR1 to stress granules under conditions of oxidative or IFN-induced stress [136]⁠.

Zα binding to Z-RNA is also biologically relevant. Z-RNA, as opposed to Z-DNA, has ribose 2′OH groups. Z-RNA formation requires more energy than that of Z-DNA. However, even with higher energy requirements, Z-RNA exists in measurable quantities in both the cytoplasm and nucleus [125]⁠. The ability for Zα to bind Z-RNA is essential for maintaining proper editing efficiencies of certain substrates [127]⁠. Indeed, the editing efficiency of ADAR1 is greatly enhanced at least in vitro by the substrates’ ability to form Z-RNA. So, Zα may contribute to the selectivity of ADAR1 for substrates [128]⁠. An example of the functionality of Zα binding to Z-RNA is ADAR1s the hyper-editing of RNA viruses. RNA viral infection can result in the triggering of the interferon pathway, and ADAR1p150 is known to be controlled by an IFN-inducible promoter [10]⁠. ADAR1p150 uniquely has a CRM1-dependent nuclear export signal that overlaps with the Zα domain and is necessary for the cytoplasmic localization of ADAR1p150 [95]⁠. RNA viruses like measles virus replicate in the cytoplasm and often form duplex RNA intermediates, so a proposed role of Zα binding is to target Z-RNA of RNA viruses resulting in hyper-editing and viral inactivation [137,138,139]⁠.

In contrast with the clear importance of Zα, the functionality of Zβ is less understood. Between ADAR1 sequences, the Zβ domain remains more highly conserved than those of the Zα domain [123]⁠. This leaves to question, if Zβ does not function similarly to Zα, then what important role does it play? The X-ray crystal structure of Zβ supported a conserved interface for metal binding including residues Glu301 and Cys304 or a large dimerization surface, but this interface may exist only as a result of crystallization conditions [123,140]⁠. While there is no clear function determined for the Zβ domain, the structural differences between Zα and Zβ explain the Zβs’ lack of Z-DNA/RNA binding.

### 6.2. Structural Comparison between the Zα and Zβ Domain

**Figure 6:**
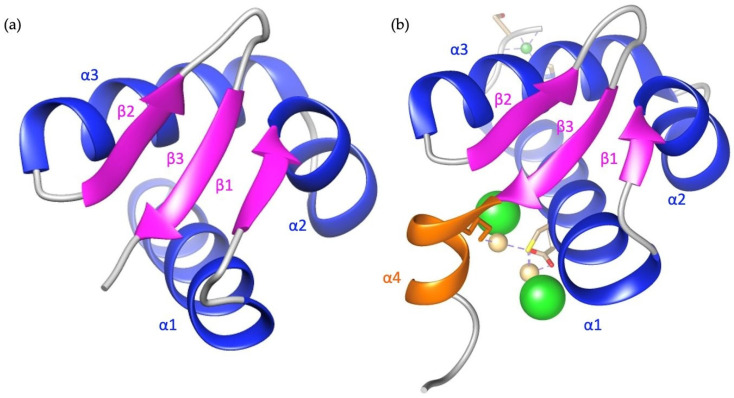
Structures of (**a**) ADAR1 Zα (PDB: 1QBJ) and (**b**) Zβ domains (PDB: 1XMK). α helices are in blue, β-strands are in magenta, the additional α helix in the ADAR1 Zβ domain is colored orange, Cd in tan, Cl in green, and Ni in yellow.

**Figure 7:**
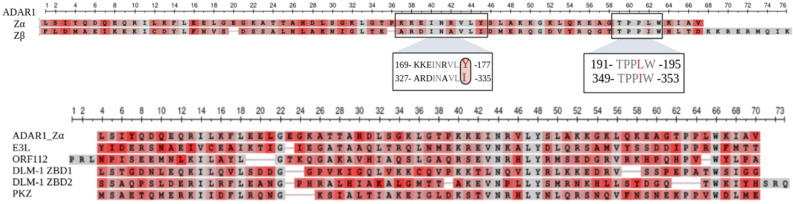
Sequence alignment of ADAR1 Zα and Zβ domains and sequence alignments of ADAR1 Zα domain with the Z-DNA binding domains of other proteins including E3L, ORF112, DLM-1, and PKZ. Color scale is column quality score. The column quality score color scale ranges from gray, highly conserved residues, to red, lowest conservation scores, and in between are intermediate scores that fall between gray and red.

The Zα domain spans residues 133–199 only present at the amino terminus of ADAR1p150, whereas the Zβ domain spans residues 293–357. The Zα domain has an α1β1α2α3β2β3 topology otherwise called a helix-turn-helix β-sheet fold (α+βHTH fold) (Figure 6a) [140]⁠. NMR structures support that in this topology the three almost perpendicular alpha helices surround a hydrophobic core up against a β2β3 hairpin [141]⁠. Residues of β2 (185–189) and β3 (194–198) form an antiparallel β sheet at the C-terminus, and the β1 sheet lies across the β2β3 hairpin with stabilizing backbone hydrogen bonds with the β3 sheet contacting Thr156, Lys194, and Trp195 [141]⁠. The α+βHTH fold contains an additional β-sheet as compared to the helix-turn-helix (HTH) motif that occurs in other proteins that recognize B-DNA [141,142]⁠. The X-ray crystal structure of the Zβ domain of ADAR1 has been captured with a resolution of 0.97 Å (Figure 6b) [123]⁠. The structure of the Zβ domain is similar to that of the Zα domain, but there is an additional alpha helix creating the topology α1β1α2α3β2β3α4 otherwise called a winged-HTH motif (Figure 6b) [123]⁠. Residues 356–363 in the additional helix, α4, are well conserved among Zβ domains [40]⁠. The α4 helix is positioned by salt bridges made between Arg362 and Lys358 with Asp342 of the β2 sheet [143]⁠. Additional contacts at the C-terminal end of α4 are made between Met363 and Phe308 and Leu355 [143]⁠. The Zβ domains of ADAR1 are highly conserved, including residues 316–324 (of β2) and 337–345 (of β3) for the tight turns of the β2β3 hairpin [143]⁠. However, between hADAR1Zα and hADAR1Zβ, only Leu318, Gly323, and Gly183 remain conserved between those ranges (Figure 7). As mentioned, the Zβ domain is unable to bind Z-DNA or Z-RNA. I will begin by describing the method of Zα binding to Z-DNA/Z-RNA and then highlight the key structural differences leading to the Zβ non-binding activity.

Structures of the Zα domain of ADAR1p150 with Z-DNA and Z-RNA have been captured [142,144]⁠. In the NMR structure of Zα in complex with Z-DNA, it was recognized that Zα has a prepositioned structure to bind Z-DNA and, upon binding, has minimal conformational alterations [141]⁠. During the conversion of B-DNA to Z-DNA, two B-Z junctions form maintaining helix strength and precise base stacking increases the stability of these junctions. Furthermore, stability can be additionally aided by Zα binding [145]⁠. The Zα domain binding to Z-DNA or Z-RNA has specificity for the Z-conformation arising from the differences in the Z versus B-shaped helix [140]⁠.

The Zα domain recognizes the Z-DNA helix on a continuous interface through the α3 helix and β2β3 hairpin (with the exception of Gly183, Gly190, and Lys196) [140]⁠. This interaction is reliant on the recognition of five adjacent phosphate residues in the sugar-phosphate backbone and stabilizing polar bonds [143]⁠. Distinctly, B-DNA binders recognize an interface with the major groove of B-DNA [146]⁠. The conservation of the interactions between the Zα domain and Z-DNA helix of other DNA and duplex Z-RNA sequences support the specificity of the Zα domain for the shape of the Z-conformation, which is sequence independent [147]⁠. Interacting residues in the hydrophobic core include Ile143, Leu147, Leu165, Ile172, Val175, Leu176, Leu179, Leu185, and Trp195 [148]⁠. Residues Ile143 and Leu144 of α1 interact with α3 through residues Ile172, Leu176, and Leu179 [141]⁠. Mutation studies support that the Ile143 and Phe146 of α1 and the Leu161 and Leu165 of α2 are necessary for the tight compaction of the two helices [141]⁠. Z-DNA contacts include Lys169, Lys170, Asn173, Arg174, Tyr177, Thr191, Pro192, Pro193, and Trp195 [148]⁠. Lys169, Asn173, and Trp195 are needed for the coordination of two ordered waters between Zα and the Z-DNA backbone and, with the addition of Tyr177, are important for Z-DNA backbone recognition [143,148]⁠. The Lys170 and Arg174 of α3 form interactions with the ribose sugar of the nucleic acid [143]⁠. Other important and highly conserved residues include Thr191, Pro192, Pro193, and Trp195. Pro192 and Pro193 are involved in van der Waals interactions with the Z-DNA and generate a rigid structure of the β2β3 loop [141,143]⁠. Mutations of these residues lead to a reduction in protein stability and an increase in proteolytic degradation [149]⁠. The Zα domain is unique to ADAR1p150 and does not appear in the other ADAR family members, but the Zα domains appear in other proteins E3L, ORF112, DLM-1/ZBP1 and PKZ [135,136,150]⁠. Within these proteins, residues Gly151, Asn173, Leu176, Tyr177Y, and Trp195 are completely conserved (Figure 7). Highly conserved residues between these proteins also included Ile143, Leu147, Val (or hydrophobic) 172, Leu179, Pro192, and Ile/Leu197. The conservation of these residues supports their importance for structure and/or function of the Zα domain.

The complementarity of the contacts described to the zig-zag sugar-phosphate backbone of the Z-DNA supports the Zα domain of ADAR1s’ shape-specificity for the Z conformation. However, the most important residue for the functionality of the Zα domain, and lack of Z-DNA/Z-RNA binding of the Zβ domain, is Tyr177. In Zβ, this crucial tyrosine of Zα becomes an isoleucine, Ile335. In comparison studies of the Zα and Zβ domains, the overall structures remain similar enough that both should conformationally recognize and be able to bind Z-DNA [148]⁠. The necessity of Tyr177 can be seen in a construct of a Zβ domain, in which all residues for the hydrophobic core interaction and Z-DNA binding contacts with the exception of Tyr177 were present as in Zα, and this protein did not bind the Z-DNA [150]⁠. Upon point mutation of Ile335 to Tyr177, Z-DNA binding activity was reported [147]⁠. Tyr177 allows for the recognition of guanine in a syn conformation, a specific trait for Z-DNA, by generating a tight van der Waals interaction to stabilize contact with the eighth carbon of the guanine [143,148,150]⁠. Trp178 further stabilizes this interaction by forming a second van der Waals interaction with Tyr177 [132]⁠. Thereby, for Z-DNA binding, the Z binding domain must recognize the unique shape of Z-DNA as well as a guanine in the syn conformation.

### 6.3. The Arginine-Rich Domain of ADAR3

ADAR3 and a splice variant of ADAR2 contain an arginine-rich domain or R motif [63,119]⁠⁠. The sequence similarity between the R domain of ADAR3 and the proposed R motif of the ADAR2 variant is closely aligned. Key residues in the R domain are lysines and six highly conserved arginine residues. In ADAR3, the R domain is required for binding to certain dsRNA and ssRNA substrates including GluR-B and 5-HT2CR RNA [63]⁠. This ADAR2 R domain-containing variant has been found to reside primarily in the cerebellum, similar to ADAR3 which, as mentioned, is restricted to the brain. Meaningfully, the R domain of both the ADAR2 isoform and ADAR3 seem to function for single-stranded RNA binding [60,116]⁠⁠. The ability for the R domain to confer ssRNA binding may act as a method for ADAR3 or this special variant to recognize substrates in vivo. The R domain has additionally been shown to mediate interactions necessary for nuclear localization [102]⁠. ADAR3 can bind in vivo to importin alpha 1 (KPNA2) through its R domain [99]⁠. The ADAR2 variant with the putative R-like motif interacts differently with importins than regular ADAR2 variants, arguing that the motif may be important for nuclear import regulation and the localization of this specific subset of ADAR2 [99]⁠. More knowledge of the tissue-specific regulation of ADAR variants is needed. Also, the ability for ADAR3’s nuclear localization to be controlled through the R domain may be a method for regulating ADAR1 and ADAR2. This arises from the fact that ADAR3 has been shown to regulate both ADAR1 and ADAR2 activity [60]⁠. Thereby, small molecules that bind to the R domain may modulate the nuclear localization of ADAR3, resulting in potential changes in ADAR1 and ADAR2 activity. There is a severe lack of knowledge in this area, so the off-target results of such modulations would be hard to gauge at this point. Other proposals for ADAR regulation suggest that within a cell or tissue type, first ADAR2 import would occur through the controls of KPNA1 or 3, then, as a mechanism of regulation increased, the KPNA2 expression may increase the ADAR3 nuclear import, resulting in the increased inhibition of ADAR1 and ADAR2 editing [99]⁠.

## 7. Future Explorations for Therapeutics and Drug Design

**Figure 8:**
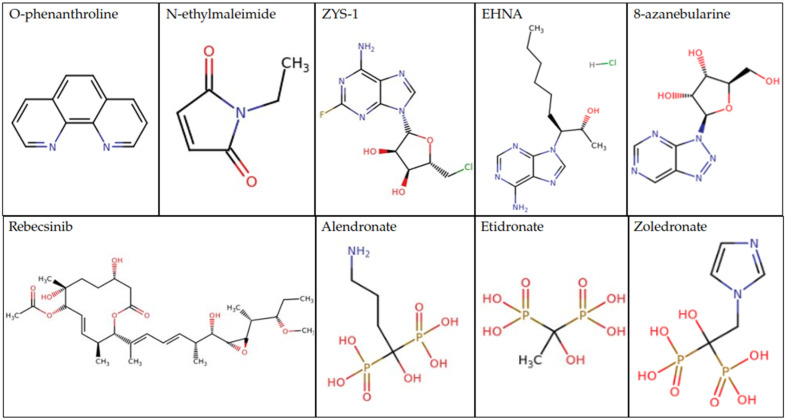
Compound scaffolds of current ADAR inhibitors [157,159,163]⁠.

Even with the leaps and bounds of ADAR structure and knowledge, there remain many avenues left to be explored to understand the complex nature of ADARs. ADARs each have their own common and unique domains, each leading to their own particular roles. This leaves much to be learned about targeting these domains for ADAR modulation. For example, Fluoxetine (Prozac), generally prescribed for depression, was shown in mice to alter the ADAR editing of the 5-HT_2C_R, the inverse of what was seen in the victims of suicidal depression [151]⁠. This indicates that the efficacy of Prozac may result from its impact on ADAR1 editing. Many current drugs for the treatment of neurological disorders have unknown mechanisms of action as the disorders themselves are often not entirely understood, leaving to question whether these too may interact with ADARs [152,153,154,155]⁠. There is a scarcity of ADAR modulators that would be useful against the extensive complications in which ADARs are involved. Therefore, producing new ADAR modulators could potentially generate new drugs and therapies for many different fields. Known ADAR inhibitors include O-phenanthroline, a zinc chelator, and N-ethylmaleimide (NEM), an alkylating reagent, both inhibitors of hRED1 [156]⁠, ZYS-1, a new inhibitor of ADAR1 that shows promise as a therapeutic in prostate cancer [157]⁠, Rebecsinib, an inhibitor of splicing mediated ADAR1 activation [158]⁠, and 8-azanebularine which inhibits ADAR2 [159]⁠ (Figure 8). Also reported to inhibit ADAR1 is erythro-9-(2-hydroxy-3-nonyl) adenine hydrochloride (EHNA) and compounds alendronate, etidronate, and zoledronate which inhibit the Zα domain of ADAR1p150 (Figure 8) [160,161]⁠. Other studies have suggested new small molecule inhibitors against the CDD IHP binding site of ADAR2 [61]⁠, the RNA binding loop of ADAR2 [162]⁠, and the Zα domain of ADAR1 [161]⁠. New insights into ADAR dimerization have also developed ADAR1 mutants that disrupt ADAR1 dimer formation and can alter the editing of particular ADAR substrates [121]⁠.

Further detailing the dimerization, protein-protein interactions, and cell/tissue-specific regulation of ADARs will be incredibly beneficial for the design of ADAR drugs and therapies that may regulate ADAR expression, localization, and the overall editing activity of specific sites. For example, the use of protein-protein interactions in regulating ADAR has been described in the editing of neuronal targets through the RNA-independent interaction with a zinc finger RBP [164]⁠. RNA binding proteins (RBPs) can alter ADAR gene expressions, and different cell types have different RBPs which can influence ADAR protein-protein interaction profiles, altering RNA editing levels in a cell-specific manner [165]⁠. Other therapeutic usages stem from understanding the crucial roles different ADARs play. For example, ADAR1 is known to be involved in recognizing self versus non-self dsRNA, a crucial part of preventing the overstimulation of dsRNA sensing pathways that could cause an intense autoinflammatory response [47]⁠. New therapies with specific ADAR1 knockout in tumor cells, where an intense response is wanted, indeed resulted in sensitization of the tumors to immunotherapy and a strong innate immune response [166]⁠. The potential for ADAR modulators in cancer spans several fields including lung, colorectal, pancreatic, breast, and prostate cancer among others [167,168,169]⁠. A field of interest within ADAR therapeutic advances is the use of ADARs in biotechnology to generate new therapies for site-specific RNA editing. These therapies rely on targeting ADAR to a specific site by either using some target mechanism like an antisense guide RNA for endogenous ADAR or guiding ADAR fusion proteins [170,171,172,173,174]⁠. Currently in phase 1 clinical trials is the drug WVE-006, which utilizes ADAR to edit a single base in the mRNA of the SERPINA1 Z allele to treat Alpha-1 Antitrypsin Deficiency (AATD) (ClinicalTrials.gov Identifier: NCT06186492). There are a variety of developments focused on site-directed RNA editing that are reviewed in the following references [175,176]⁠.

Notably, there are some limitations to ADAR therapeutics. ADAR small molecule inhibitors must be highly specific and site-directed ADAR systems need optimized guide RNAs to reduce off-target editing. ADARs play a wide range of roles, and currently, there are still questions about the breadth of ADAR pathways. As key RNA editors, manipulation of their editing levels and off-target editing can result in adverse reactions. There is a risk that in aiming to affect one disease target, another disease mechanism may be triggered. Recently, research in prostate cancer using the ADAR1 inhibitor, ZYS-1, has supported that ADARs can be druggable targets with favorable safety profiles, at least in the tested prostate cancer models [157]⁠. Results so far support that ZYS-1 inhibition of ADAR1 can significantly reduce prostate cancer proliferation, invasion, and metastasis [154]⁠. Other ADAR therapies like site-directed ADAR editing can have many other limiting factors that are still being overcome, including the low stability of RNA. These therapies can be immunogenic, and the delivery systems are extremely important in overall therapeutic efficacy [176]⁠. In trying to overcome these limitations, there has been success in using circular RNAs to increase guide RNA stability, alteration of the guide RNAs to avoid the foreign RNA sensor mechanisms and reduce immunogenic response, and increased knowledge in tissue-specific interactions and variants that can help increase therapeutic editing efficiency [177,178,179]⁠.

Overall, ADARs are significant proteins for a wide variety of roles related to human health and as structure often dictates function, the knowledge of ADAR structures provided here is highly relevant for future drug design and discovery.

## Figures and Tables

**Table 1:** Key residues within the ADAR2 catalytic deaminase domain. A visualization of this table is provided in Supplementary Figure S1.

Key Structural Points for ADAR2 Catalytic Domain	Residues
Zinc Coordination	H394, E396, C451, and C516.
IHP Coordination	Coordination Residues: N391, R400, R401, K518, R522, W523, K629, Y658, K662, Y668, Q669, K672, W687, E689, and D695.
	Side Chain Contacts: D392, I397, L511, M514, V688, and K690.
	Hydrogen Bond Involvement: K483.
RNA Interface/Contact Residues	Coordination Residues: R348, V351, T375, K376, E396, Q488, C451, E455, R481, S486, T490, S495, R510, and K594.
	Coordination Residues in RNA Binding Loop: N473, R474, K475, and R477.
RNA Binding Loop	A454, R455, I456, F457, S458, P459, H460, E461, P462, I463, L464, E465, E466, P467, A468, D469, R470, H471, P472, N473, R474, K475, A476, and R477.
Base Flipping Loop	G487, Q488, and G489.

**Table 2:** Summary table of key contact residues within the ADAR2 dimer model including contacts of the substrate binding monomer (CDD 1) with the dsRNA, the contact between the dsRBD of the editing monomer and dsRNA, and the contacts between the two CDD of ADAR2.

Key Structural Points in Homodimer Structure	Residues
Contacts between CDD 1 and dsRNA	Backbone Contacts: T375, C451, K475, S486, T490, and K594.
	Side Chain Contacts: T375, K376, E396, R455, H460, R474, R477, R481, S486, Q488, R510, and K594.
Contacts between dsRBD-2 and dsRNA	Backbone Contacts: N235, S258, and K281.
	Side Chain Contacts: N235, E242, S258, H259, R279, N280, K281, and K282.
Dimerization Helix	T501, W502, D503, G504, V505, L506, Q507, G508, and E509.
Protein–Protein Contacts in CDD 2	G452, R455, F457, S458, H460, R481, Q488, T490, R590, and G593.

## Supplementary Materials

The following supporting information can be downloaded at: https://www.mdpi.com/article/10.3390/cimb46050243/s1.
