# Supplementary material for: ADAR Family Proteins: A Structural Review
Source: Curr Issues Mol Biol. 2024 Apr 26;46(5):3919–45. doi: 10.3390/cimb46050243 (PMC11120146; doi:10.3390/cimb46050243)
Supplement: Supplementary file 1 [file cimb-46-00243-s001.zip › cimb-2938527-supplementary.pdf]

Supplementary Information
ADAR Family Proteins: A Structural Review

Figure S1. Visualization for Table 1, PDB: 5ED2 (ADAR2 CDD mutant E488Q bound to GLI1 dsRNA sequence). The CDD of ADAR2 is presented in cyan. Nucleic acid database (NDB) color scheme was used for the dsRNA. In blue are the zinc coordination residues, these 4 amino acids are labeled. In pink are the residues that facilitate IHP coordination within the active site. In red are the coordination residues within the RNA interface or contact residues. In orange are the residues that make up the RNA binding loop. In gray are the residues of the base flipping loop.
Figure S2. Sequence alignments of dsRBDs for each ADAR family member using constraint-based multiple alignment tool (COBALT). Color scale is column quality score. The column quality score color scale ranges from gray, highly conserved residues, to red, lowest conservation scores, and in between are intermediate scores that fall between gray and red.
Figure S3. Structure of one monomer from PDB: 7ZLQ (in magenta) bound to dsRNA (NDB color scheme). Conserved residues from sequence alignments in Figure S2 are highlighted in blue.
Table S1. Summary table of key contact residues within the ADAR1 dimer model including contacts within the β-sheet interface and the contacts from the dsRBDs to dsRNA.

Supplementary Figures

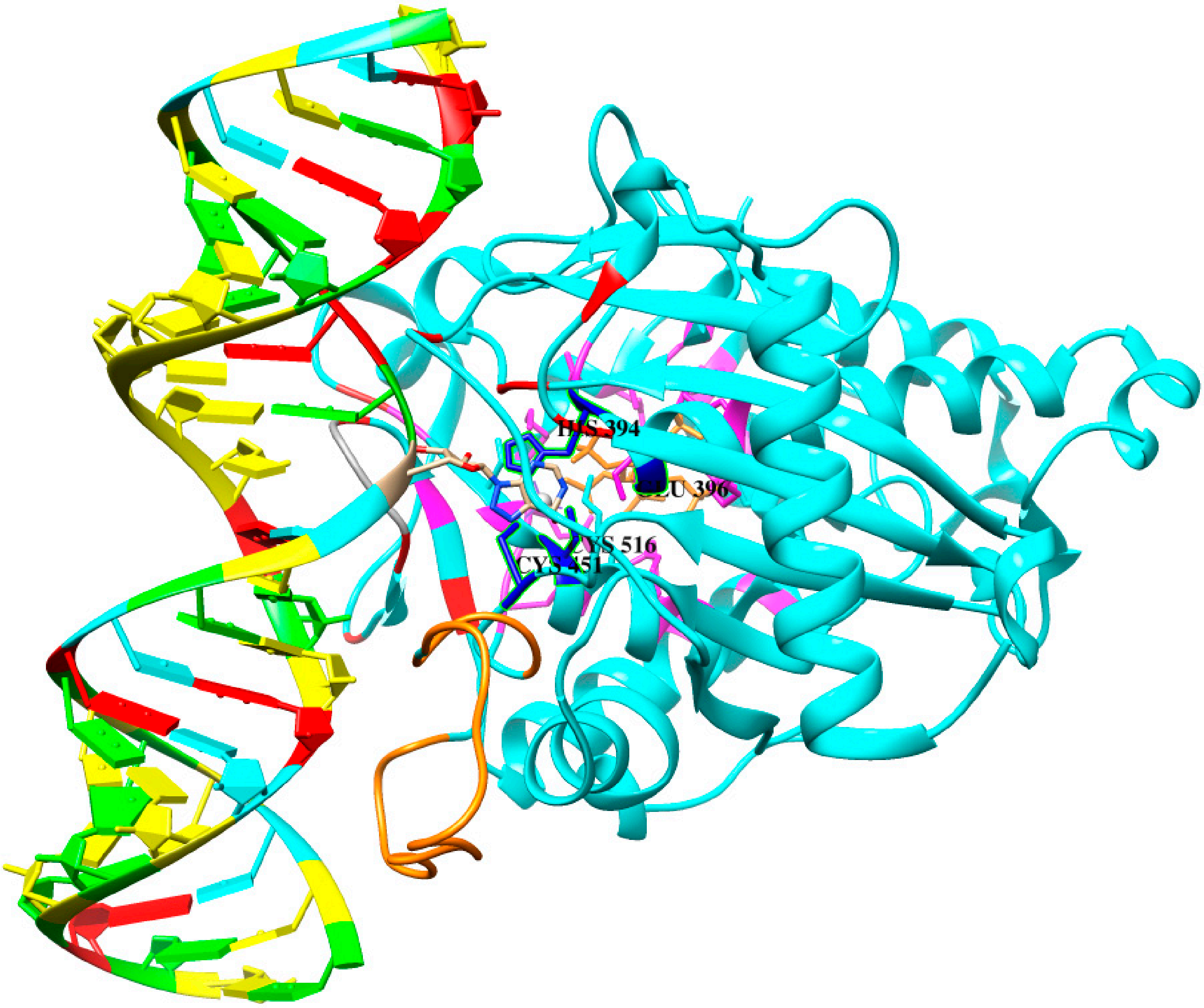

Figure S1. Visualization for Table 1, PDB: 5ED2 (ADAR2 CDD mutant E488Q bound to GLI1 dsRNA

sequence). The CDD of ADAR2 is presented in cyan. Nucleic acid database (NDB) color scheme was used for the dsRNA. In blue are the zinc coordination residues, these 4 amino acids are labeled. In pink are the residues that facilitate IHP coordination within the active site. In red are the coordination residues within the RNA interface or contact residues. In orange are the residues that make up the RNA binding loop. In gray are the residues of the base flipping loop.

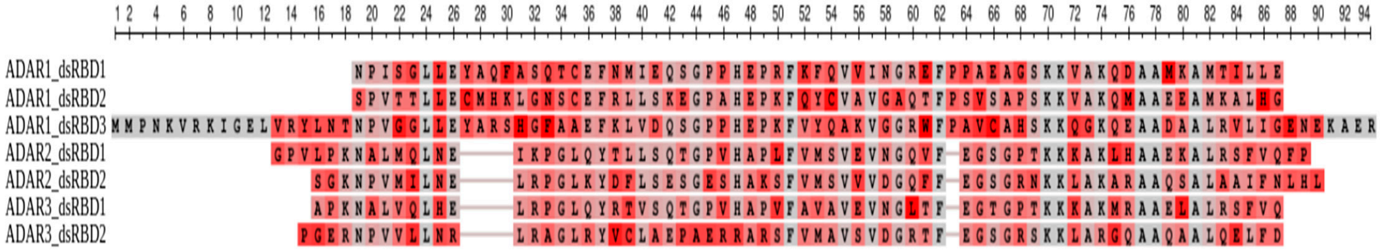

Figure S2. Sequence alignments of dsRBDs for each ADAR family member using constraint-based multiple alignment tool (COBALT). Color scale is column quality score. The column quality score color scale ranges from gray, highly conserved residues, to red, lowest conservation scores, and in between are intermediate scores that fall between gray and red.

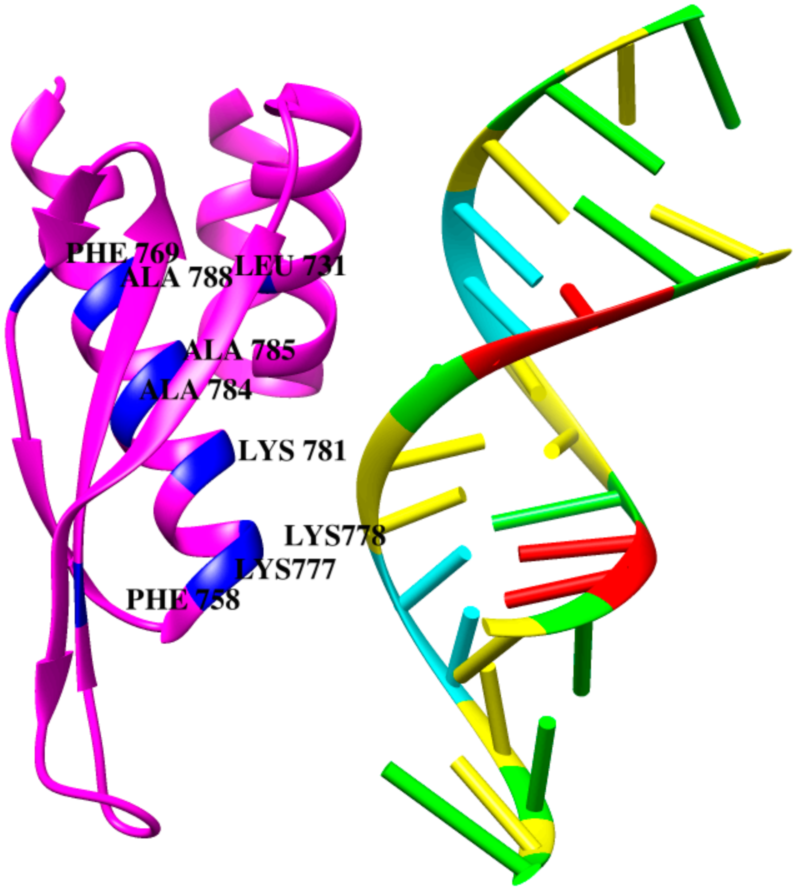

Figure S3. Structure of one monomer from PDB: 7ZLQ (in magenta) bound to dsRNA (NDB color scheme). Conserved residues from sequence alignments in Figure S2 are highlighted in blue.

Supplementary Table

Key Structural Points in Homodimer Structure	Residues
β-Sheet Interface	β1: V747 and D748.
	β2: K757, V759, and Q761.
	β3: W768, P770, A771, and C773.
3dsRBD dsRNA Contacts	αN: R721 and N726.
	α1: E733 and R736.
	β1- β2 loop: P753 and H754.
	α2: K777, K778, and Q782.

Table S1. Summary table of key contact residues within the ADAR1 dimer model including contacts within the β-sheet interface and the contacts from the dsRBDs to dsRNA.
